# The phase transition of Pb_8_F_14_I_2_

**DOI:** 10.1007/s00706-016-1854-z

**Published:** 2016-10-28

**Authors:** Matthias Weil

**Affiliations:** Division of Structural Chemistry, Institute for Chemical Technologies and Analytics, TU Wien, Getreidemarkt 9/164-SC, 1060 Vienna, Austria

**Keywords:** Lead fluoride halide, Crystal structure determination, Thermal analysis, Group–subgroup relationships

## Abstract

**Abstract:**

The reversible phase transition of Pb_8_F_14_I_2_ is of continuous type and takes place at about 107 °C as monitored by temperature-dependent single crystal and powder X-ray diffraction measurements, optical microscopy, and differential scanning calorimetry. The low-temperature ferroelastic phase crystallizes in the orthorhombic crystal system (23 °C, *Bmmb*, *Z* = 2, *a* = 6.0699(6) Å, *b* = 6.0165(6) Å, *c* = 25.077(2) Å, 1487 structure factors, 41 parameter, *R*(*F* ^*2*^) = 0.0346, *wR*(*F* ^2^) = 0.0771) and changes its symmetry to the tetragonal crystal system into the high-temperature paraelastic phase (130 °C, *I*4/*mmm*, *Z* = 1, *a* = 4.2667(12) Å, *c* = 25.388(7) Å, 430 structure factors, 303 parameter, *R*(*F* ^*2*^) = 0.0575, *wR*(*F* ^2^) = 0.1564). Group–subgroup relationships between the two structures and a hypothetical intermediate structure are presented.

**Graphical abstract:**

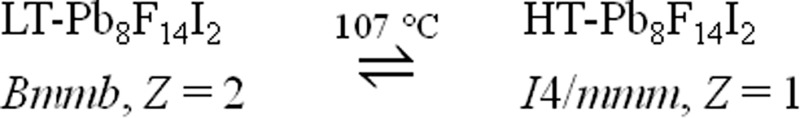

## Introduction

In the pseudo-binary system PbF_2_/PbI_2_, the phases (PbF_2_)_7_(PbI_2_), (PbF_2_)_5_(PbI_2_), and (PbF_2_)_4_(PbI_2_) have been synthesized in the form of single crystals and their crystal structures reported by Aurivillius [1]. Synthesis and unit cell parameter of polycrystalline matlockite-type [2, 3] PbFI have been described by Rulmont [4] and Aurivillius [5]; the crystal structure of the latter phase was refined afterwards from single crystal data [6].

The crystal structure of (PbF_2_)_7_(PbI_2_) has originally been determined by Aurivillius on the basis of integrated room temperature Weissenberg data in the orthorhombic space group *Bmmb* (standard setting *Cmcm,* No. 63) with lattice parameters *a* = 6.0711(6) Å, *b* = 6.0198(5) Å, and *c* = 25.084(2) Å [1]. For that purpose single crystals were originally grown by boiling and recrystallizing a sample with nominal composition of 10PbF_2_·PbI_2_ in water. The author reported a systematic twinning of the very tiny and thin crystals in the way that the *a*- and *b*-axes are interchanged. Due to the method of data collection, the platy crystal form and clearly visible twinning of the investigated single crystal, a straightforward structure refinement was hampered at that time, and the reliability index of 0.109 based on |*F*| was rather high. However, all atomic positions could be derived, and for Pb and I atoms anisotropic temperature factors were considered in the final model. The nearly equal lengths of the *a*- and *b*-axes and an *I*-centred pseudo-tetragonal subcell make it appear likely that a phase transition from the room-temperature modification (low-temperature (LT) phase) into a tetragonal high-temperature modification (high-temperature (HT) phase) occurs. Although the author of the original study has theoretically derived an idealized structure model in space group *I*4/*mmm* with the unit cell relations: *a*_ideal._ ≈ (*a*_LT_ + *b*_LT_)/√8; *c*_ideal._ ≈ *c*_LT_, neither experimental details regarding a possible phase transition nor structure data of the assumed high-temperature phase were reported at that time or afterwards. Therefore, a more detailed examination of a possible phase transition and a redetermination of the crystal structure of (PbF_2_)_7_(PbI_2_) (= Pb_8_F_14_I_2_) seemed worthwhile.

In this article preparation, ferroelastic behaviour, phase transition and crystal structures of Pb_8_F_14_I_2_ at room temperature (LT modification) and at 130 °C (HT modification) as well as their relationships are reported.

## Results and discussion

### Phase transition

**Fig. 1:**
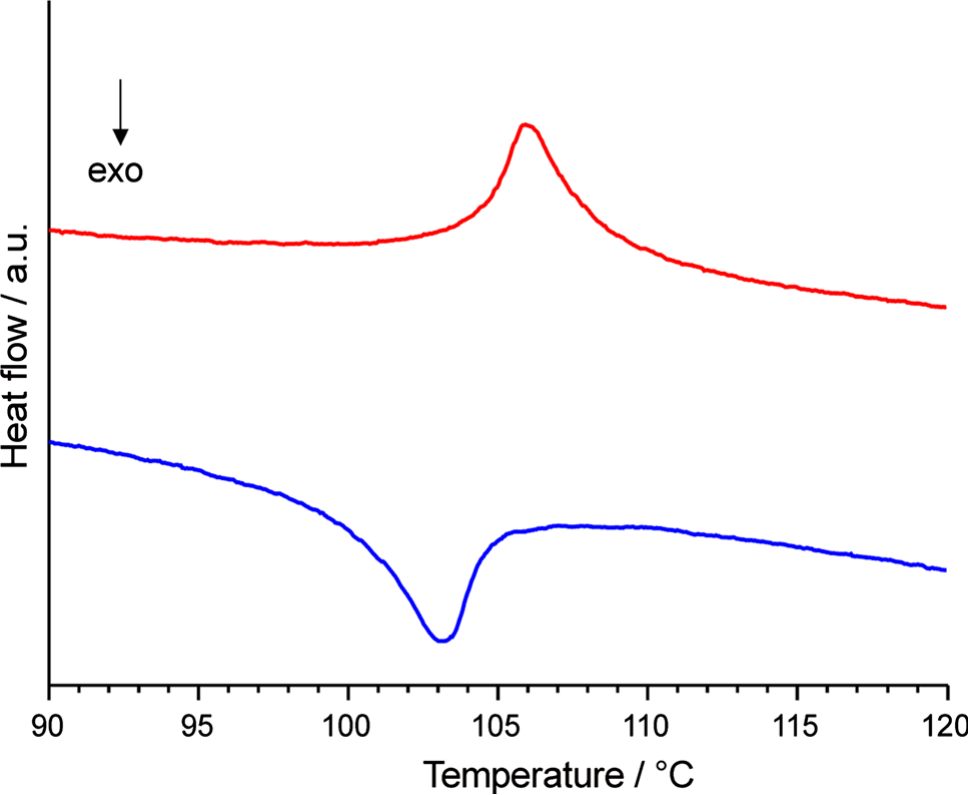
DSC curves obtained during heating (*red*) and cooling (*blue*) of Pb_8_F_14_I_2_ (colour figure online)

Examination of Pb_8_F_14_I_2_ (LT) single crystals under a microscope in transmitted polarized light revealed clearly visible multiple domains for most of the crystals, in accordance with the observations by Aurivillius [1]. The domain crystals show ferroelastic behaviour [7] and can be partially or completely reorientated by application of stress with a pair of tweezers along the diagonal or parallel to the *a*- or *b*-axes (in the setting of space group *Bmmb*). By heating single crystals in inert silicon oil on a Kofler heating stage, a spontaneous change from a biaxial to an uniaxial crystal system in terms of the change of the birefringence of crystal faces was observed at 107(2) °C, indicating a phase transition from the orthorhombic to the tetragonal crystal system. This process was reversible and showed virtually no hysteresis. The observed transition temperatures are in good agreement with a complementary DSC measurement, with on-set temperatures of 104.6 °C (heating; endothermal effect) and 104.0 °C (cooling; exothermal effect) (Fig. 1).

**Fig. 2:**
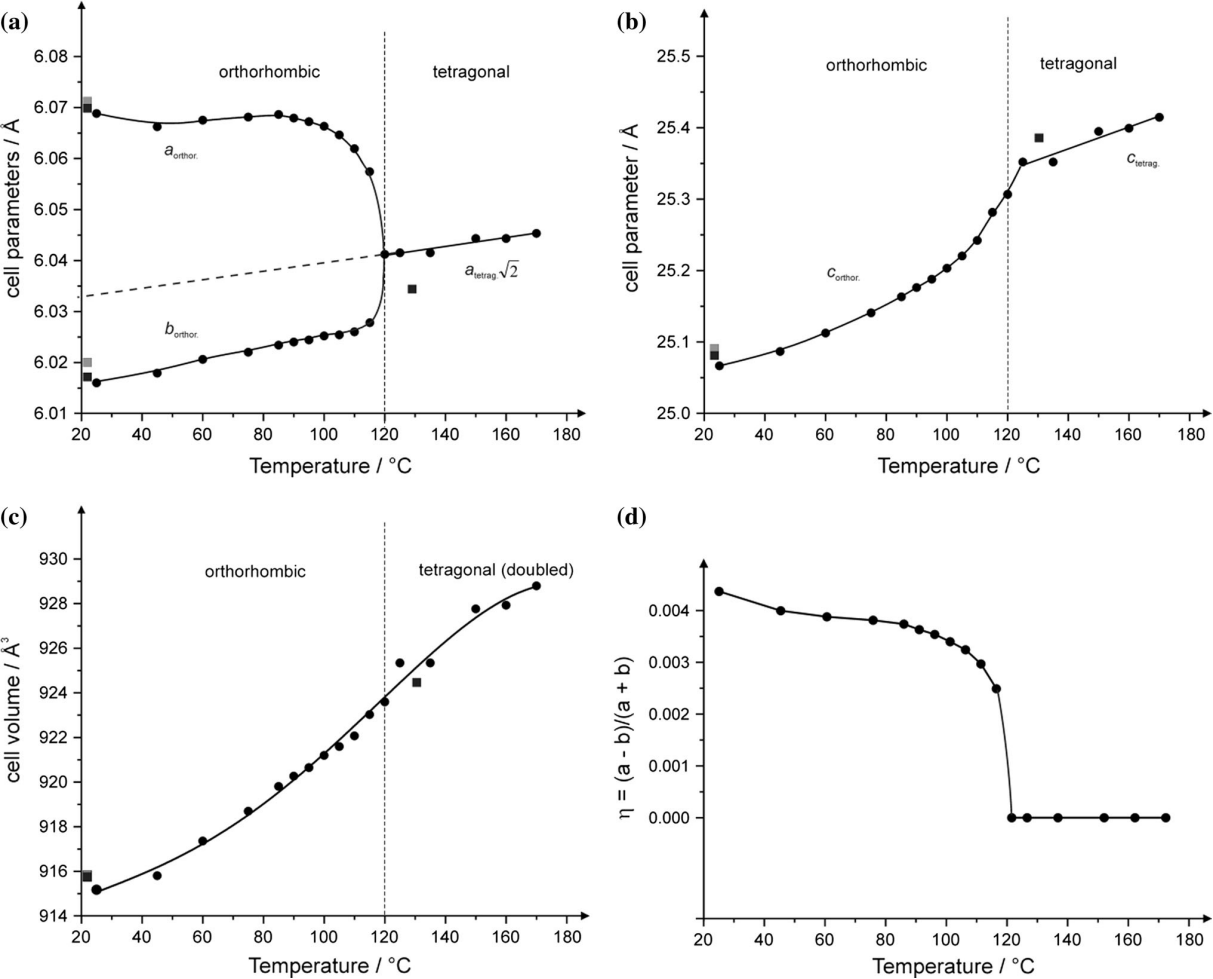
Evolution of unit cell parameters of Pb_8_F_14_I_2_ with temperature. The crystal has tetragonal symmetry at *T* > *T*_C_ and orthorhombic symmetry at *T* < *T*_C_, with *a* > *b*; single crystal diffraction data from Aurivillius (*grey squares*) [1], current single crystal diffraction data (*black squares*), powder X-ray diffraction data diffraction (*black circles*); the size of the symbols is greater that the standard deviation of individual values. **a** *a* and *b* axes (the identical unit cell parameters *a* and *b* in the tetragonal HT-phase can be extrapolated into the temperature interval of the orthorhombic LT-phase, *dashed line*); **b** *c* axis; **c** volume; **d** plot of the temperature-dependent course of the order parameter *η* of Pb_8_F_14_I_2_

The temperature-dependence of the unit cell parameters for the LT- and HT-phases, as evidenced by X-ray diffraction measurements, is given in Fig. 2. The derived transition temperature of the diffraction experiments is somewhat higher (118(8) °C) for this kind of measurement than those from the optical examinations or DSC measurements. This behaviour is ascribed to the more inaccurate temperature measurement during the diffraction studies where the temperature sensing device was displaced a couple of millimetres from the actual sample. Given the different temperature sensing for the three methods, the derived transition temperature from the DSC experiment appears to be that with the highest accuracy.

The evolution of the orthorhombic unit cell parameters *a* and *b* (Fig. 2a) reveals a more obvious change of *a* towards the transition point than that of *b*. The *c* unit cell parameter appears not to be directly involved in the transition mechanism (Fig. 2b), with a non-linear behaviour for the temperature-dependence of the LT-phase and a linear behaviour for the HT-phase. The resulting change of the cell volume with temperature is depicted in Fig. 2c. The ratio (*a* − *b*)/(*a* + *b*), where *a* and *b* are the lengths of the orthorhombic unit cell at a given temperature, is a suitable order parameter *η* for classification of the type (continuous, discontinuous) of the phase transition. The development of the temperature-dependence of *η* = (*a* − *b*)/(*a* + *b*) is given in Fig. 2d and indicates a continuous phase transition from orthorhombic to tetragonal symmetry upon heating. This classification is supported by the continuous change of the unit cell volume with temperature close to the transition point and the observation of the very slight hysteresis observed during optical examination of crystals at the Kofler stage in polarized light or in the DSC curve (Fig. 1) during heating and cooling above and below the transition point.

**Fig. 3:**
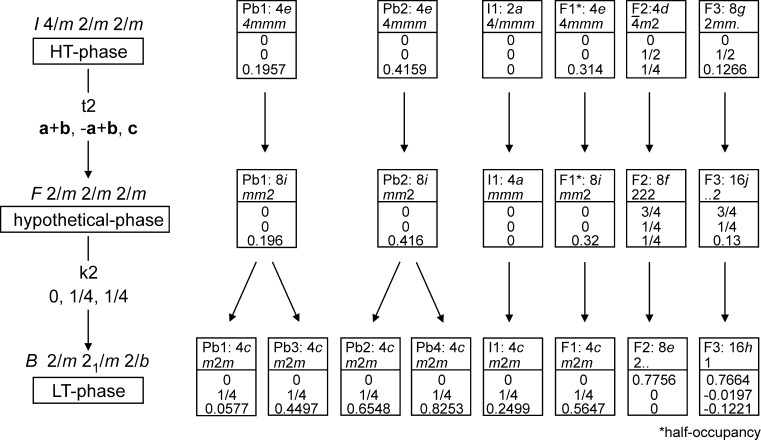
Group–subgroup relations for the HT- and LT-forms of Pb_8_F_14_I_2_ and the hypothetical intermediate phase in *Fmmm*. Coordinates of HT- and LT-Pb_8_F_14_I_2_ refer to the refined coordinates (deposited as CIFs, see *Experimental*) but here due to truncation with somewhat lower precision

Above the transition temperature Pb_8_F_14_I_2_ is tetragonal, representing the paraelastic phase in space group *I*4/*mmm*; below the transition temperature it is orthorhombic, representing the ferroelastic phase in space group *Bmmb*. There is no direct group–subgroup relation between these two space groups [8, 9], because *Bmmb* (or in its standard setting *Cmcm*) is not a maximal subgroup but a general subgroup of *I*4/*mmm* with *Fmmm* being the intermediate space group type. The group–subgroup relationships of the paraelastic (HT) and ferroelastic (LT) structures of Pb_8_F_14_I_2_, together with the hypothetical intermediate structure model in *Fmmm*, are presented in the form of a Bärnighausen family tree [10] in Fig. 3.

The symmetry reduction from *I*4/*mmm* to *Fmmm* is of *translationengleiche* (t) kind with an index of 2, accompanied by a loss of the fourfold rotation axes present in the higher-symmetry structure. The relationship between the *Fmmm* structure and *Bmmb* is of *klassengleiche* (k) kind with an index of 2 under change of the Bravais centering from *F* to *B*. The first *translationengleiche* transition of index 2 from *I*4/*mmm → Fmmm* is a ferroic transition [11] and explains the presence of two twin domains in the resulting crystals of the LT-Pb_8_F_14_I_2_ polymorph.

### Structure description: LT-phase

**Fig. 4:**
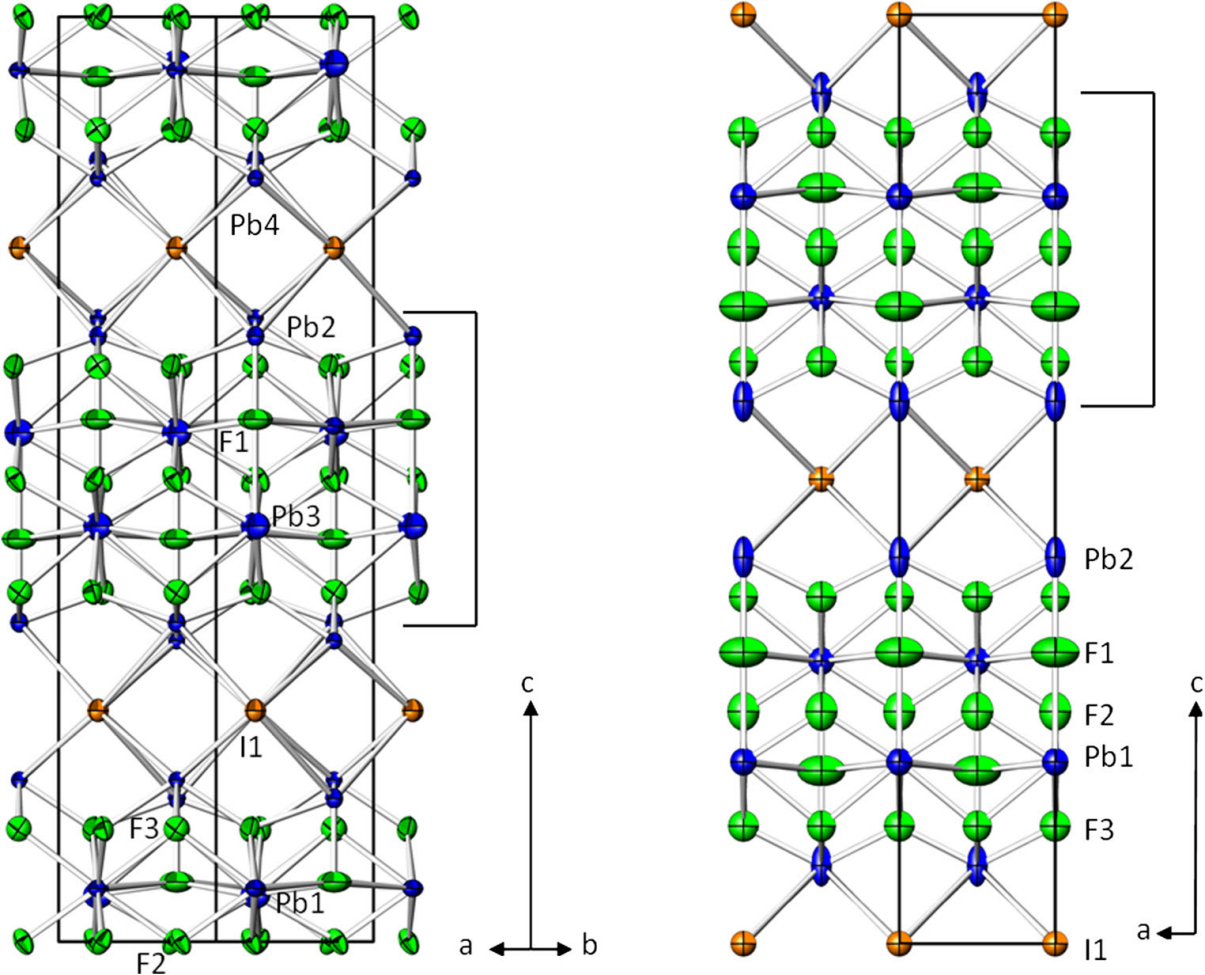
The crystal structures of Pb_8_F_14_I_2_ in the LT-(*left*) and HT-(*right*) modifications, with projections along [110] and [010], respectively. Anisotropic displacement parameters are drawn at the 74% probability level. The *right brackets* designate the “PbF_2_”-type blocks in the structure

The current structure refinement confirms the original model [1]; however, with increased precision and accuracy. The crystal structure is made up of four-layer “PbF_2_”-type blocks consisting of four unique Pb^2+^ cations. The blocks are stacked along [001] with a mean Pb–F distance of 2.63 Å which is in good agreement with the sum of the ionic radii [12] for Pb^2+^ and F^−^ (2.60 Å; weighted according to the different coordination numbers as discussed below). The “PbF_2_”-type blocks are separated by interstitial I^−^ ions that are located at *z* ≈ ¼ and ¾ (Fig. 4, left) with a mean Pb–I distance of 3.70 Å. The “PbF_2_”-type blocks contain additional ordered F^−^ ions (F1) with one close contact of 2.261(10) Å to Pb2. The latter defines one of the boundaries of the blocks and is additionally bonded to the interstitial I^−^ ions. Due to the additional F1 ions, the coordination numbers (CN) of the central Pb^2+^ cations in each block are increased in comparison with fluorite-type PbF_2_ (CN = 8), to CN = 10 for Pb1 (resulting coordination polyhedron: bicapped cube) and CN = 11 (tricapped cube) for Pb3. The terminal Pb^2+^ cations at the boundaries of the blocks exhibit CN = 9 for Pb2 (four I^−^ and four F^−^ ions plus one capping F^−^; distorted monocapped square antiprism), and CN = 8 for Pb4 (four I^−^ and four F^−^ ions; distorted square antiprism). All Pb–F and Pb–I bond lengths (Table 2) are comparable with those of matlockite-type PbFI [6] or the other structures in the system PbF_2_/PbI_2_ [1].

### HT-phase

In the structure of the HT-phase (Fig. 4, right) only two unique Pb^2+^ cations are present; the interstitial F1 atom now shows half-occupancy. The central Pb^2+^ cation (Pb1) of the “PbF_2_”-type block has CN = 11 (tricapped cube) and the terminal Pb^2+^ cation (Pb2) has CN = 9 (four I^−^ and four F^−^ ions plus one capping F^−^; monocapped square antiprism). In comparison with the structure of the LT-phase, the mean Pb–F distance in the “PbF_2_”-type blocks slightly increases to 2.70 Å, whereas the mean Pb–I distance of the LT-phase and that of the HT-phase are more or less the same. Comparison of the individual Pb–F and Pb–I bond lengths reveals a shift of individual atoms up to 0.25 Å for the lighter F and 0.15 Å for heavy I atoms.

## Experimental

### Preparation

Single crystals of Pb_8_F_14_I_2_ were grown under hydrothermal conditions from stoichiometric amounts of PbI_2_ (Riedel-de Haën, pure) and PbF_2_ (Aldrich, 99 + %). The starting materials were homogenized by grinding and placed in a 5-cm^3^ Teflon^®^ container that was filled up to two-thirds of its volume with demineralized water. The Teflon^®^ container was then closed and placed in a steel autoclave and subjected to the following heating protocol: 25 → 250 °C [2 h], 250 °C [10 days], 250 → 25 °C [10 h]. Nearly colourless laminated crystals of Pb_8_F_14_I_2_ and few very thin crystals with a plate-like form and light-yellow colour of PbFI [6] were obtained. Microcrystalline samples of Pb_8_F_14_I_2_ were prepared by reaction of stoichiometric amounts of PbF_2_ and PbI_2_ in sealed and evacuated silica ampoules at 380 °C for 1 week.

### DSC measurements

DSC measurements were performed with ~20 mg samples on a NETZSCH DSC 200F3 system in the temperature range 30–150 °C (aluminium crucibles with pierced lid, flowing argon atmosphere (20 cm^3^/min), heating rate 5 °C min^−1^).

### X-ray diffraction and single crystal structure analysis

Polycrystalline samples were characterized by X-ray powder diffraction with Cu*K*_α1,2_ radiation (*λ* = 1.54060, 1.54439 Å). At room temperature a Philips X’pert system was used. Temperature-dependent measurements were performed on a Philips PW1012/10 diffractometer equipped with a home-built heat controlling system. Unit cell parameters were refined with the program TOPAS [13].

Single crystal X-ray data were collected on a Bruker APEX II CCD diffractometer with MoK_α_ radiation (*λ* = 0.71079 Å). For single crystal X-ray measurements at room temperature, optically controlled single domain crystals of Pb_8_F_14_I_2_ (RT, 23 °C) were fixed with cyanoacrylate adhesive on thin silica glass fibres. For high-temperature measurement of Pb_8_F_14_I_2_ (HT, 130 °C), a more temperature-resistant two-component adhesive was used. Heating was provided with a gas-flow heater using a stream of nitrogen. Diffraction data for the two measurements were corrected for absorption effects with the program HABITUS [14]. Full-matrix least-squares refinements on *F* ^2^ for the two data sets and correction of extinction effects were carried out with the program SHELXL-2014 [15].

For the LT-phase the coordinates of the reported structure model [1] were taken as starting parameters for refinement in the space group *Bmmb*. The measured crystal was twinned by pseudo-merohedry with a rotation by 90° along [001] as twin element (refined twin ratio of the twin domains 1:1). For the HT-phase the proposed coordinates of the idealized structure model [1] in the space group *I*4/*mmm* were used. For the two models anisotropic displacement parameters for all atoms were refined.

Details of data collections and structure refinements are listed in Table 1. Selected bond lengths are collated in Table 2. Further details of the crystal structure investigations may be obtained from Fachinformationszentrum Karlsruhe, 76344 Eggenstein-Leopoldshafen, Germany (fax: (+49)7247-808-666; e-mail: crysdata@fiz-karlsruhe.de, https://www.fiz-karlsruhe.de/icsd.htm) on quoting the appropriate CSD number listed at the end of Table 1. Drawings of structural details were produced using the program ATOMS [16].

**Table 1:**
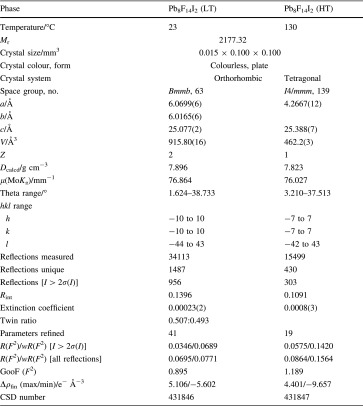
Details of single crystal X-ray data collections and structure refinements

**Table 2:** Selected bond lengths/Å

Pb_8_F_14_I_2_ (LT)			Pb_8_F_14_I_2_ (HT)		
Pb1–F2	2.492(5)	4×	Pb1–F2	2.5404(9)	4×
Pb1–F3	2.693(6)	4×	Pb1–F3	2.762(11)	4×
Pb1–F1	3.0400(7)	2×	Pb1–F1	3.00(6)	
Pb2–F1	2.261(10)		Pb1–F1	3.027(5)	4×
Pb2–F3	2.434(5)	4×	Pb2–F3	2.390(8)	4×
Pb2–I1	3.8415(9)	2×	Pb2–F1	2.59(6)	
Pb2–I1	3.8594(9)	2×	Pb2–I1	3.6967(14)	4×
Pb3–F2	2.579(6)	4×			
Pb3–F3	2.789(6)	4×			
Pb3–F1	2.884(10)				
Pb3–F1	3.0298(13)	2×			
Pb4–F3	2.381(5)	4×			
Pb4–I1	3.5504(8)	2×			
Pb4–I1	3.5756(8)	2×			
